# Biofilm Mode of Cultivation Leads to an Improvement of the Entomotoxic Patterns of Two *Aspergillus* Species

**DOI:** 10.3390/microorganisms8050705

**Published:** 2020-05-11

**Authors:** Frédéric Francis, Florent Druart, José Diana Di Mavungu, Marthe De Boevre, Sarah De Saeger, Frank Delvigne

**Affiliations:** 1 Functional and Evolutionary Entomology, TERRA Research Center, Gembloux Agro-Bio Tech, University of Liege, 5030 Gembloux, Belgium; fdruart@student.ulg.ac.be; 2 Microbial Processes and Interactions (MiPI), TERRA Research Center, Gembloux Agro-Bio Tech, University of Liege, 5030 Gembloux, Belgium; 3 Centre of Excellence in Mycotoxicology and Public Health, Faculty of Pharmaceutical Sciences, Ghent University, 9000 Ghent, Belgium; jose.dianadimavungu@ugent.be (J.D.D.M.); Marthe.DeBoevre@UGent.be (M.D.B.); Sarah.DeSaeger@ugent.be (S.D.S.)

**Keywords:** *Aspergillus flavus*, *Aspergillus oryzae*, secondary metabolites, *Culex quinquefasciatus*, submerged culture, biofilm, mycotoxins, biological control, entomopathogenic microorganism

## Abstract

Two fungi, i.e., *Aspergillus flavus* Link and *Aspergillus oryzae* (Ahlb.) E. Cohn, were cultivated according to two methodologies, namely submerged and biofilm cultures with the primary aim to use their secondary metabolites the supernatant CL_50_, and CL_90_ varied between 1.3% (*v/v*) to 12.7% (*v/v*) for incubation times from 24 to 72 h. While the *A. flavus* supernatant entomotoxicity was higher than this of *A. oryzae*, the biofilm culture application increased the efficiency of the former. Proteomic analysis of the supernatants revealed discrepancies among the two species and modes of cultivation. Furthermore, the secondary metabolite profiles of both *Aspergillus* cultures were verified. Aspergillic acid, beta-cyclopiazonic acid, cyclopiazonic acid, ferrineospergillin, flavacol, and spermadin A were most predominant. Generally, these secondary metabolites were present in higher concentrations in the supernatants of *A. flavus* and biofilm cultures. These molecular identifications correlated positively with entomotoxic activity. Noteworthy, the absence of carcinogenic aflatoxins was remarkable, and it will allow further valorization to produce *A. flavus* to develop potential biopesticides.

## 1. Introduction

About 700 fungal species are listed as insect biocontrol agents, with many of them belonging to the order of Hypocreales, such as *Beauveria* and *Metarhizium* [1]. These fungi penetrate through the outer integument. During the spore germination phase, enzymes are produced to allow for the development of the germ tube. Infectious filaments penetrate the cuticle through mechanical and enzymatic action and allow fungi to grow and exert their toxicity against some insects [2]. *Aspergillus sp.* is a well-known fungus that is used for diverse industrial applications and it was demonstrated to have entomotoxic effects against mosquitoes and aphids. Different *Aspergillus* species were found in the environment of the insect, but only a few have a direct insecticidal effect [3]. The most frequently encountered species are *A. flavus* and *A. parasiticus* residing on different insects (*Apis mellifera carnica, Hyblaea puera*). Two hypotheses were proposed regarding the physical mode of action of *Aspergillus*, i.e., either (1) the mycelium grows after direct contact on the insect cuticule or (*2*) the mycelium is ingested and proliferates into the insect gut. The latter is consistent with the mechanism of action of the Hypocreales [4] (Seye et al., 2014). Besides the physical mode of action, some *Aspergillus*, such as *A. flavus,* produce lytic enzymes and secondary metabolites. However, some of them, such as aflatoxin, are carcinogenic and detrimental for both animal and human health [5,6] (WHO-WHO-IARC, 2002; Woloshuk & Shim, 2013). The assessment of aflatoxin presence in strains is then of utmost importance, as non-toxigenic strains are proposed as biopesticides to control insect pests [7,8,9].

Spores of *A. flavus* and *A. clavatus* were observed to reach virulence levels comparable to *M. anisopliae*, which is a well-known fungus used in biological pest control. The spores can be easily produced at a high density (10^9^ conidia/g) in a semi-solid medium on an inexpensive substrate based on wheat bran. The compounds produced by *Aspergillus*, which may be involved in the insecticidal activity, are mainly secondary metabolites, including enzymatic proteins. Medina and colleagues [10] pinpointed the influence of the composition of the culture medium on the excretion of proteins by *A. flavus*. Half of the proteins identified were enzymes that were involved in protein or carbohydrate catabolism. In another study, Kang and colleagues [11] showed that a basic liquid medium provided a maximum production of α-galactosidase by *A. parasiticus*. Additionally, *Aspergillus* fungi produce secondary metabolites that act as a chemical defense against fungal predators. These include insecticidal compounds, such as tetramic acids, indole terpenoids, pyridones, and diketopiperazines [12].

Different modes of cultivation can be considered for filamentous fungi. Semi-solid fermentation (SSF), where the fungi grow on a solid medium as a biofilm, allows for the production of interesting secondary metabolites in higher amounts [13]. Indeed, it seems that the secretion of metabolites is promoted in SSF. However, these systems exhibit several technical limitations, such as the low mass and heat transfer coefficients and the difficulties for product recovery from the solid substrate. Better control of the bioreactor operating conditions can be achieved in conventional submerged cultures. However, in submerged culture, the filamentous fungi tend to form aggregates (pellets), possibly reaching several centimeters in diameter and leading to a heterogeneous physiological state. Additionally, effective growth only occurs at the surface of the mycelial aggregate due to nutrient limitation in the central region [14].

An interesting trade-off between the biological enhancement of metabolites production due to mycelium attachment and optimal control of bioreactor operating conditions can be achieved in biofilm reactors. In this system, fungal biofilm is self-attached on an inert surface (e.g., stainless steel) and it is continuously or sequentially immersed in a liquid medium [15,16]. Such a biofilm reactor design allows for combining the advantages of the culture in semi-solid medium and the control of the operating conditions of the submerged culture, consequently allowing for easier recovery of secondary metabolites [16]. A variety of substances (exopolysaccharides, proteins, nucleic acids, surfactants, lipids, glycolipids, and cations) are excreted to form a polymeric matrix. Finally, the cells detach and disperse to form a new biofilm [17]. In addition, this biofilm formation technology can be exploited on a pilot or semi-industrial scale for the effective production of secondary metabolites by combining the aforementioned advantages of semi-solid and submerged cultures. A continuous nutrient circulation system provided oxygen and nutrients in order to produce and enhance the quality of excreted proteins and secondary metabolites. A study by Seye and colleauges [4] showed that the secondary metabolites that were produced in biofilms by *A. flavus* were more effective against mosquitoes than the ones obtained based on submerged cultures. Different hypotheses have been put forward regarding the mode of action and the production of toxic secondary metabolites in entomotoxic fungi with regards to the production methodology [4]. Here, we aimed to investigate the active fungal entomotoxic compounds that were based on proteome and secondary metabolome analysis, broadcasting different toxicological efficiency. Particular attention will be paid on the impact of the bioreactor system used for the production of metabolites, i.e., either submerged or biofilm cultivation mode.

## 2. Materials and Methods

### 2.1. Mosquito Breeding

*Culex quinquefasciatus* Say mosquito, S-lab strain, was reared in a controlled room at 25 ± 2 °C, 70 ± 5% relative humidity, and a 16 h light photoperiod in 50 × 50 × 50 cm net cages (Bug Dorm, Megaview Science, Taiwan). They were fed with sponges that were impregnated with a 10% sucrose solution. Female adults were also exposed three times a week to blood meals for reproduction using a Hemotek^®^ membrane feeding systems (Hemotek Ltd., Blackburn, UK) and heparinated bovine blood from University experimental farm. Egg rafts were daily manually collected and maintained in 25 (length) × 15 (width) cm containers with 5 (depth) cm distilled water. After hatching, the larvae were fed every two days with a powder of 2:1 crushed fish food (Tetramin^®^, Belgium) mixed and natural brewer’s yeast (Biover^®^, Belgium). Water was renewed every week. The rearing conditions were 25 ± 2 °C temperature, 70 ± 5% relative humidity, and 16:8 h (light:dark) photoperiod.

### 2.2. Aspergillus Cultivation

**Figure 1:**
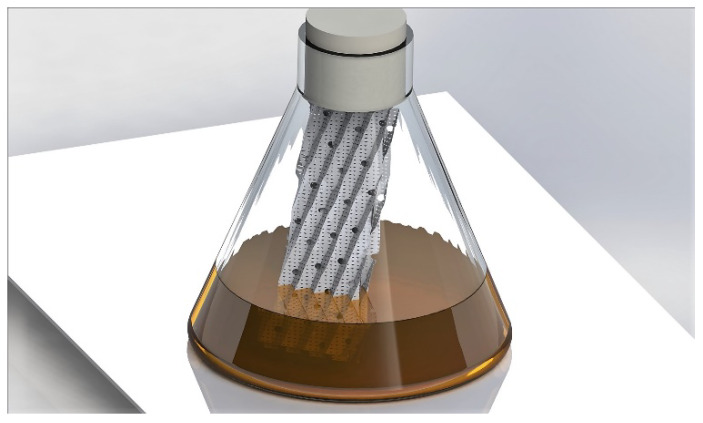
Illustration of the biofilm cultivation device used in this study. The system comprises a standard, non-baffled, shake flask with two stainless steel sheets for promoting the attachment of the fungal biomass.

*A. flavus* (accession number MUCL 55276) was isolated from the larvae of *Agriotes lineatus* (L.) (Coleoptera: Elateridae). The strain of *A. oryzae* originated from a transgenesis between *A. oryzae* ATCC16868 *pyrG* [18] and a plasmid pAB4.1 as a selection marker source [19]. Both species of *Aspergillus* were cultivated in the same mode. The strains were cultured on a potato dextrose agar medium (PDA) in a kneaded dish at 30 °C. The conidia were suspended by scraping the fresh cultures in distilled water with 10% tween 80 and 8.5 g/L NaCl. The cultures were carried out in 250 mL Erlenmeyer flasks (121 °C for 20 min), each containing 100 mL of yeast extract peptone dextrose (YPD) medium. The detailed composition was 10 g/L of yeast extract, 10 g/L of casein peptone and 20 g/L of glucose. The whole medium was autoclaved at 121 °C for 20 min. before inoculation of the conidia with approximately 10^6^ conidia/100 mL of medium. The strains were cultured in two modes: (1) in submerged conditions in conventional shake flasks and (2) in biofilm formation conditions in shake flasks that were equipped with a partially immersed support. This support was composed of two pieces of porous stainless steel 316 L (70 mm × 60 mm, Sulzer, Winterthur, Switzerland) that were arranged in opposition and attached (Figure 1). The same system has been previously used for growing *Trichoderma harzianum* for the production of hydrophobin [20] and for the growth of *Rhizopus oryzae* in interaction with yeasts and lactic acid bacteria [21]. The cultures were performed at 30 °C and 125 rpm for five days.

Sampling procedures for biomass dry weight determination were applied on both cultivation modes. For the submerged culture, the culture was first filtered over a MN615 127 filter (MACHEREY-NAGEL^®^, Germany) and the filtrate was subsequently filtered over a sterile 0.22 μm pore size syringe filter (Sigma-Aldrich Millex^®^ GV, Germany) to recover all of the spores and mycelial filaments. The biomass collected on the 0.22 μm filter was used for biomass weight determination. In the biofilm cultivation device, the biomass grew exclusively on the packing elements. Therefore, the determination of the biomass dry weights in biofilm cultures was performed by removing the solid support from the flasks. Upon removal, the support materials were kept dried for 2 h at room temperature to remove excess liquid. For both the submerged and biofilm culture, the biomass dry weight was finally determined by gravimetry after drying the filter/support for 24 h at 105 °C.

### 2.3. Acute Toxicity Test

Toxicity tests to assess the short-term effects were conducted according to the methods that were recommended by the World Health Organization [22]. Only filtrates of microbial biomass were used and diluted in distilled water. The conditions of temperature, humidity, and light were similar to those of the rearing, namely 25 ± 2 °C, 70 ± 5% RH and 16 h light period. The applied concentrations (*v/v*) were from 1% to 10%. For each test, distilled water was included as the control. Twenty larvae of *Cx. quinquefasciatus* 3rd instar were placed in 50 mL of fungal dilutions. Three replicates were performed for each tested dilution. The dead larvae were counted and removed from the medium after 24 and 48 h of exposure.

### 2.4. Proteomic Analysis

The supernatants of both *Aspergillus* species that were produced from two culture conditions, and a negative control (i.e., medium) were filtered using Vivaspin ultrafiltration (Sartorius^®^, Goettingen, Germany) at a molecular weight of 10 kDa to remove the unconsumed peptides from the culture medium. The concentrated proteins were then suspended in a solution of 100 mM phosphate buffer (pH 6.5). The quantification of the proteins of each sample was performed while using the RC DC Protein Assay kit (Bio-Rad, Hannover, Germany). Protein extracts (25 μg samples, three replicates/sample) were labeled with cye-dyes (GE Healthcare, Diegem, Belgium) according to the standard 2D-DIGE protocol. Protein samples were subjected to pairwise comparison. Two fungal protein samples from two different species/culture mode, labeled with Cy3 or Cy5, were mixed with an internal reference. The internal reference was made from pooled aliquots from all experimental samples labeled with Cy2. A conventional dye swap for 2D-DIGE was performed. A supplementary protein (125 μg from each *Aspergillus* production) was added to form a protein mixture for a preparative gel on which the protein spots were taken for identification analysis.

The first-dimensional isoelectric focusing was performed for 2D-DIGE. The mixture of labeled proteins was adjusted to a volume of 450 μL and was used to rehydrate 24 cm immobilized pH gradient (IPG) strips (pH 3–10 NL) (GE Healthcare, Diegem, Belgium) for 12 h at 20 °C at a constant voltage (50 V). Isoelectric focusing (IEF) was carried out at 200 V for 200 Vh, 500 V for 500 Vh, 1000 V for 1000 Vh, and 8000 V for 60,000 Vh at 20 °C. A maximum current setting of 50 μA/strip was used in an isoelectric focusing unit (BioRad, Hannover, Germany). Subsequently, after IEF, the IPG strips were equilibrated for 15 min. in 375 mM Tris (pH 8.8) containing 6 M urea, 20% (*v/v*) glycerol, 2% (*w/v*) sodium dodecyl sulphate (SDS), and 130 mM 1,4-Dithio-DL-threitol (DTT). Afterwards, IPG strips were incubated for 15 min. in the same buffer without DTT containing 135 mM iodoacetamide. These IPG strips were then placed in contact with a two-dimensional (2D) HPE NF 12.5% Wide Gel (Serva, Germany). The proteins on the IPG band then migrated on the gel according to their molecular weight through a high-performance electrophoresis (HPE) FlatTop Tower (Gelcompany, Heidelberg, Germany). Finally, the gels were put in a solution of 15% ethanol and 10% acetic acid for 4 h to fix the proteins. The gels were scanned with an Ettan DIGE Imager fluorescence scanner (GE Healthcare) at exposures that corresponded to each CyDye. The images were analyzed with Progenesis SameSpots (Nonlinear Dynamics, Newcastle, UK).

Protein spots with significant differences (*p* < 0.05) between fungal species and culture mode were selected for further analysis. The spots were picked from the preparative gel while using a Screen Picking system (Proteomic Consult, Kampenhout, Belgium). Each excised spot was placed in a well (96-well plate) and proteins were trypsin-digested on a Janus workstation (Perkin Elmer). Briefly, the gel pieces were washed with three incubations in 100% of 50 mM ammonium bicarbonate, and a mix of 50% acetonitrile 50% of 50 mM ammonium bicarbonate. Two additional washes were performed with 100% acetonitrile to dehydrate the gel. Freshly activated trypsin (Roche, porcine, proteomics grade) was used to rehydrate the gel pieces at 8 °C for 30 min. Trypsin digestions were performed for 3 h at 30 °C. Peptide extractions were performed with 10 μL of 1% formic acid for 30 min. at 20 °C.

For mass spectrometry based on a MALDI-TOF-TOF-MS Ultra-Xtreme (Bruker), automatic spectral acquisition was performed with Flex control™ v3.4 software control and real-time analysis by Flex analysis™ v3.4 (Bruker, Germany). The database search was managed in real time with BioTools™ v3.2 (Bruker) on the Mascot v2.2.06 server. The searches were performed with the SwissProt database on fungi with 100 ppm mass error tolerance. A second search was performed with the same data on the NCBI database without taxonomic restriction. Protein scores were considered to be sufficient when they reached 45 or more. The pI and the molecular weights of the suggested proteins were compared with the location of the spots excised on the gels. A search with BLAST (Basic Local Alignment Search Tool) was performed to refine the identification of several proteins.

### 2.5. Metabolomic Analysis

Ten mL of samples were placed in a 50 mL falcon tube. Fifteen mL of acetonitrile (ACN) were added before being vortexed for 2 min. A mixture of salts (4 g of MgSO_4_ and 1 g of NaCl) was added, the tube was vigorously shaken by hand for 1 min. and then vortexed for 2 min. Subsequently, the samples were centrifuged at 4500 rpm for 5 min. and 5 mL of the supernatant was transferred into a glass tube. The samples were evaporated to dryness under a gentle nitrogen stream and were then resuspended with 250 μL of MeOH/ACN/H_2_O (30/30/40, *v/v/v*) and centrifuged in an Ultrafree-MC^®^ centrifugal tube for 5 min. at 14,000 g. Finally, the extract was transferred into an LC-MS/MS vial.

The chromatographic separation was performed by a FTN UPLC class 1 system (Waters, Manchester, UK) using a ZORBAX RRHD Eclipse Plus C18 column (1.8 μm, 3.1 × 100 mm). The mobile phase consisted on H_2_O/MeOH (99/1, *v/v*) containing 0.05% HCOOH and 5 mM HCOONH_4_ (Solvent A) and MeOH (Solvent B). A gradient elution program was applied, as follows: 0–0.5 min.: 5% B, 0.5–20 min.: 5–95% B, 20–21 min.: 95% B, 21–24 min.: 95–5% B, 24–28 min.: 5% B. The flow rate was 0.3 mL/min. The temperature of the column was set at 40 °C and the temperature of the autosampler was 10 °C. Five µL of the sample were injected.

Mass spectrometry (MS) analyses were performed while using a Synapt G2-Si HDMS quadrupole hybrid high-definition MS (Waters, Zellik, Belgium), equipped with an electrospray ionization source (ESI). The data were acquired in positive (ESI^+^) and negative (ESI^−^) mode in resolution mode (> 20,000 FWHM). The MS parameters were: capillary voltage (2.8 kV, ESI^+^ and −1.5 kV, ESI^−^); sample cone voltage (30 V); source temperature (150 °C); desolvation gas flow (800 L/h) heated to a temperature of 550 °C; and, a cone gas flow rate (50 L/h). Nitrogen was used as a desolvation gas. Argon was used as a collision gas at a pressure of 9.28 × 10^−3^ mbar. The instrument was calibrated while using sodium formate. During MS analyses, a leucine-enkephalin solution (200 μg/μL) was continuously infused into the MS at a flow rate of 20 μL/min., generating the reference ion ([M+H]^++^ = 556.2771; [M+H] ^+^ = 554.2615) used for mass correction. Mass spectra were collected in continuous mode from 50 to 1,200 m/z with a scan time of 0.1 s, an inter-scan scan delay of 0.01 s and a lock-out frequency of 20 s. A data-dependent acquisition mode is set up to obtain simultaneous acquisition of exact mass data for the ions. The first five ions were selected for MS/MS from a single MS investigation. The scan time for MS/MS was 0.2 s. The collision energy in the trap cell was reduced from 10/15 V (low mass, start/end) to 60/150 V (high mass, start/end). Instrument control and data processing were performed while using the Masslynx v4.1 (Waters) software.

### 2.6. Statistical Analysis

Corrected mortality ratios were calculated to evaluate lethal concentrations [20] (Abbott, 1925) and were they linearized using logit transformation: log (P) = ln (P/1) – P) [23]. Linear regression was used to model the relationship between logit-transformed mortality and log-transformed values of fungal concentrations as an explanatory variable: logit (P) = slope × ln (concentration) + intercept. The relationship between larval mortality and spore concentrations was assessed based on Snedecor-F distribution and *p*-values. Statistical analyses were performed using Minitab^®^ software v.16 (http://www.minitab.com/en/default.aspx). For all tests, the significant threshold was *p* < 0.05.

## 3. Results

### 3.1. Biomass Production of the Two Species of Aspergillus

The dry matter that was produced by *A. flavus* in submerged culture averaged 1.04 ± 0.08 g per 100 mL of medium after five days, while, in biofilm, 1.16 ± 0.10 g. The yields of obtained materials were 0.52 g/g and 0.58 g/g of carbon source, respectively, since the initial medium contained 2 g of glucose per 100 mL. The dry matter produced by *A. oryzae* in submerged culture averaged 0.61 ± 0.03 g per 100 mL of medium after five days, while 0.55 ± 0.02 g in the biofilm. The yields of obtained materials were 0.30 g/g and 0.27 g/g of carbon source, respectively. Significant differences of produced biomass were observed according to *Aspergillus* species (F = 144.38 and *p* < 0.001), production way for *A. flavus* (F = 9.32 and *p* = 0.038), and for *A. oryzae* (F = 11.65 and *p* = 0.027).

### 3.2. Acute Toxicity Test of Aspergillus Supernatants

**Figure 2:**
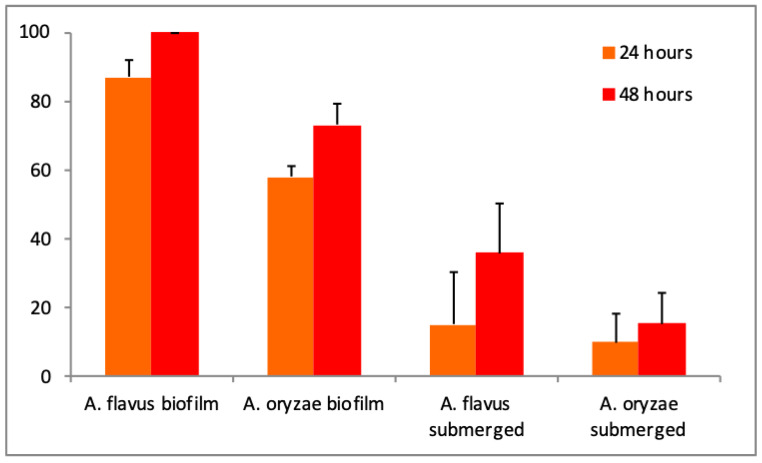
Evolution of *Culex quinquefasciatus* mortality rates according to *Aspergillus* species and production mode with a 10% supernatant dose.

Mortality rates increased with time and with supernatant concentration. The supernatant concentration to reach the highest mortality was 10% in submerged culture and in biofilm (data not shown). The maximum mortality was 36% for the submerged culture of *A. flavus*, whereas it was at least 73% for biofilm culture of *A. oryzae* after 48 h (Figure 2). Significant differences in entomotoxicity were observed according to production way (F = 99.50 and *p* < 0.001), *Aspergillus* species (F = 11.28 and *p* = 0.004), and exposition duration (F = 6.01 and *p* = 0.026).

Only the lethal concentrations (LC_50_, LC_90_) in the supernatant could be calculated for those produced in the biofilm given the low mortality rate for the submerged cultures. The calculated lethal concentrations logically decreased with the incubation time from 24 to 48 h (LC_50_–LC_90_ from 4.62 to 2.24%–12.71 to 7.20% and LC_50_–LC_90_ from 8.40 to 6.96%–15.12 to 13.98% for *A. flavus* and *A. oryzae*, respectively). Globally, the lethal concentrations differed between 1.30% (LC_50_ at 72 h for *A. flavus*) and 15.12% (LC_90_ at 24 h for *A. oryzae*).

### 3.3. Proteomic Analysis of Aspergillus Supernatants

Based on the comparison of the proteins excreted in *A. flavus* and *A. oryzae* supernatants in relation to the culture mode (submerged or biofilm), 18 spots were observed to be statistically differentially expressed by the two-dimensional electrophoresis approach (Table 1). The fold ratios of protein expression differed according to the two culture modes (biofilm and submerged) for all of the identified proteins. Eleven proteins, including some unknown/hypothetical ones, were identified with isoelectric points that ranged between 3.9 and 5.2, and molecular weights higher than 13 kDa. Four enzymes were found: a glucoamylase, a phosphohydrolase, an exonuclease, a formyl-Coa transferase, and one another protein involved in cell division. Glucoamylase and phosphohydrolase were found in *A. oryzae* supernatants. The first was found in both production modes and the second was found in the submerged culture. A protein originating from a strain of *A. oryzae* was mainly found in the submerged culture. In the supernatants of *A. flavus* in submerged culture, phosphohydrolase, formyl-Coa transferase, and exonuclease Rrp41 were found. A protein originating from a strain of *A. oryzae* was found in the submerged culture.

### 3.4. Secondary Metabolomic Analysis of Aspergillus Supernatants

In ESI^+^, out of a total of 97 potential compounds, six secondary metabolites were detected in culture supernatants (Table 2). These were identified as aspergillic acid, beta-cyclopiazonic acid, cyclopiazonic acid, ferrineaspergillin, flavacol, and speradin A. Ferrineaspergillin was not found in the *A. oryzae* supernantants. *A. flavus* excreted more diversified molecules when the fungus was grown at biofilm than in submerged culture. MS analysis in ESI^−^ revealed the presence of three of the compounds already identified in ESI^+^, namely beta-cyclopiazonic acid, cyclopiazonic acid, and speradine A. None of the other compounds that were generally more easily identified in ESI^−^ was detected.

## 4. Discussion

Fungal biomass produced after five days was different between the submerged and the biofilm mode of cultivation. Nevertheless, the yield was 1.9 times higher for *A. flavus* than *A. oryzae*, while the inoculum was identical in both cases. For *A. flavus*, the average biomass produced represented a maximal bioconversion yield. Zune and colleagues [16] produced 0.55 g/g of carbon source with *A. oryzae* in biofilm, but the medium contained starch as a carbon source, and not glucose *in casu*. In the submerged culture, the biomass formed pellets for both fungal strains.

Supernatants from submerged cultures were less toxic to mosquito larvae than those that were produced in biofilms, for both *Aspergilli*. The supernatants obtained from the biofilm cutlivations with *A. flavus* were significantly more efficient and induced higher insect mortality than those of *A. oryzae*. Several studies pinpointed the insecticidal effects of different species of *Aspergillus*, but the authors often used microbial biomass in toxicity tests. De Moraes and colleagues [24] demonstrated the efficacy of several species of *Aspergillus*, including *A. flavus* against *Aedes fluviatilis*. However, the same strains were tested on *Culex quinquefasciatus* without success, with conidial concentrations in the range of 10^5^ to 10^6^ conidia/mL. In contrast, Bawin and colleagues [2] performed toxicity tests with a spore concentration of 10^6^ conidia/mL of *A. flavus* against *Cx. quinquefasciatus*. After five days of incubation, the mortality reached 83% and the LC_50_ was calculated as 1.8 × 10^8^ conidia/mL for an incubation time of 72h. Following the discovery of a strain of *A. oryzae* XJ-1 on *Locusta migratoria*, the authors found LC_50′_s of 3.3 × 10^8^, 1.7 × 10^7^, and 7.2 × 10^6^ conidia/mL after 10, 13, and 15 days, respectively [25]. Other mortality tests that were carried out by Vijayan and colleagues [26] demonstrated the effect of 35 fungal secondary metabolites against *Cx quinquefasciatus, Anopheles stephensi* and *Aedes aegypti* with similar LC_50_ values that we obtained.

The supernatants of the submerged cultures of *A. flavus* and *A. oryzae* had higher protein contents than those in the biofilm. Khalesi and colleagues [15] explained the possibility that some molecules are trapped inside the biofilm matrix. Additionally, the protein synthesis varied according to the culture type. A similar observation was pointed out [27]. Glucoamylase was overexpressed by *A. oryzae* in both culture types, which was not surprising, as this species is known for its industrial production of amylase [28]. Another protein of *A. oryzae,* namely phosphohydrolase, was found in the supernatants. This enzyme cleaves phosphate bonds and it can interfere in the hydrolysis of ATP [29]. The phosphohydrolase was overexpressed in the submerged cultures of both *Aspergillus* species. This was identified in another entomotoxic fungus, *Beauveria bassiana*, and it was considered as insecticidal molecule [30]. Phosphatases were involved in the insecticidal role of *Bacillus thuringiensis* [31].

An additional identified protein was a formyl-CoA transferase, which catalyzes the transfer of the CoA fraction from formyl-CoA to oxalate and *vice versa*. Therefore, the enzyme is involved in the degradation of oxalate and it is generally found in acidophilic organisms [32]. This protein is overexpressed by *A. flavus* in the submerged culture. A cell division protein was also identified in our cultured fungal proteomes to be overexpressed in *A. flavus* biofilm and in *A. oryzae* submerged cultures. This might explain a higher cell division activity for biofilm cultures. Subsequently, exonuclease, which is an enzyme that cleaves nucleic acids that are present in RNA [33], was overexpressed in submerged cultures of both *Aspergilli.* The latest proteomic analysis performed in the entomology laboratory in Gembloux showed the presence of proteases that could be determinant for a potential pathogenicity (Francis, unpublished results). This family of proteins has not been observed in our supernatants while varying amounts of glucoamylase, phosphohydrolase, sulfite reductase (NADPH), formyl-CoA transferase, cell division protein, and exosome exonuclease were observed. Zune and colleagues [16] performed a proteomic analysis of the same strain of *A. oryzae* was performed by two-dimensional electrophoresis. Four families of enzymes have been identified: proteases, polysaccharide hydrolases, hydrolases of glycosyl groups, and oxyreductases. The culture medium was less rich in carbohydrate and protein sources. In addition, the authors used starch (not glucose) and the identified proteins were in the same range of the isoelectric point and molecular weight as in our analysis.

Many strains of *A. flavus* produce aflatoxins in different agricultural crops (maize, peanuts, groundnuts, and cottonseeds) and these toxins are powerful carcinogenic agents that mainly target the liver [34]. No aflatoxins were observed in the supernatants of both species of *Aspergillus*. Moreover, multiple insecticidal compounds that were produced by *Aspergillus* have been identified, such as tetramic acids, indole terpenoids, pyridones, and diketopiperazines [35] (Blaney and Green, 1989). The molecules that have been identified in our analysis are all part of these chemical families. Cyclopiazonic acid is an indole-tetramic acid that is mainly found in cereals [36,37,38]. Cyclopiazonic acid is an inhibitor of the Ca^2+^ ATP-ase pump of the sarcoplasmic and endoplasmic reticulum [39] (Nishie et al., 1985). Acute toxicity tests by oral ingestion on chicken, rabbit, dog, pig, and rat have shown gastrointestinal, neurological, and degenerative effects inducing organ necrosis (such as liver, heart, and kidney) [40]. Oral LD_50′_s were from 12 to 64 mg/kg bw depending on the animal species (mice, rats, chickens or men) (Fremy, 2009) [41]. Blaney and Green [35] performed tests on stage 1 larvae of *Lucilia cuprina* with cyclopiazonic acid. The obtained LC_50_ was determined to be 80 mg/kg. Additionally, speradine A was observed, which is a derivative of cyclopiazonic acid, also called 1-N-methyl-2-oxoCPA [42].

Aspergillic acid, ferrineoaspergillin, and flavacol are derivatives of natural pyrazines [43]. MacDonald and colleagues [44] have demonstrated the biosynthetic pathway of neoaspergillic acid: leucine flavacol, neoaspergillic acid, neohydroxyaspergillic acid. The hydroxyaspergillic acid has a structure close to aspergillic acid, and it was found in both *A. flavus* and *A. oryzae* [45]. Ferrineoaspergillin is a complex formed from aspergillic acid and Fe^3+^. These molecules derived from aspergillic acid are antibiotics that have inhibitory activities on Gram-negative, Gram-positive bacteria and on human cancer cells [46]. Finally, three mycotoxins (cyclopiazonic acid, aspergillic acid, and speradine A) were identified in our work. Leporins B and C are molecules that were composed of pyridin-2-ones moieties and they were not found in our supernatants but cited in other studies related to *Aspergillus* [47] (Arroyo-Manzanares et al., 2015). At the level of the two species studied in our work and their culture method, analysis tended to prove in our case that secondary metabolites were more present in *A. flavus* and in biofilms. Taking all of these observations into account, the level of toxicity of fungal culture supernatants could be correlated with: (1) the species of *Aspergillus*, (2) the cultivation mode. More specifically the submerged culture was associated to the production of less toxic supernatants, because the mycelium grows as pellets, leading to either more trapping or a reduced excretion of secondary metabolites, by comparison with self-immobilized free mycelia observed during biofilm cultivations. This latter mode of cultivation is particularly useful for the efficient production of entomotoxic compounds and could be further investigated for the up-scaling of such bioprocesses.

In conclusion, this work indicated the usefulness of the biofilm mode of cultivation for the production of fungal supernatants. These supernatants could be further considered to be the basis of formulations for biocontrol application.

## Figures and Tables

**Table 1:** Protein identification by LC-MS/MS from *Aspergillus flavus* and *A. oryzae* supernatants produced with two culture modes (Biofilm and Submerged).

*A. flavus* Biofilm	*A. oryzae* Biofilm	*A. flavus* Submerged	*A. oryzae* Submerged	Protein Identification	Accession Number	Mascot Score	Coverage	Molecular Weight	pI	Organisms
				glucoamylase	BDHI01000015.1	52	15	68,837	4.1	*Aspergillus awamori*
				phosphohydrolase	WP_066372067.1	84	23	24,463	5.2	*Bacillus*
				hypothetical protein	WP_027406331.1	87	31	13,278	3.9	*Anaerovibrio* sp.
										
										
										
				cell division protein	WP_062096136.1	72	31	24,637	5	*Arthrobacter* sp.
										
				sulfite reductase (NADPH)	SDC68807.1	95	22	63,455	6.1	*Aspergillus awamori*
										
				hypothetical protein	BAE63852.1	91	47	26,086	4.7	*Aspergillus oryzae*
				hypothetical protein	BAE63852.1	87	37	26,086	4.7	*Aspergillus oryzae*
										
				formyl-CoA transferase	WP_066772048.1	88	22	44,240	5	*Croceicoccus mobilis*
										
										
				hypothetical protein	WP_064318324.1	93	44	21,046	4.7	*Bibersteinia trehalosi*
				exosome exonuclease	WP_048094625.1	93	29	28,821	5.1	*Geoglobus ahangari*

Fold ratio of protein expression according to a heatmap illustration. No significant identification was obtained for empty lines. 
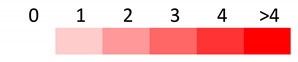

**Table 2:** Secondary metabolite identification by UPLC-TOFMS from *Aspergillus flavus* and *A. oryzae* supernatants produced with two culture modes.

Chemical Class	Name	Formule	Exact Mass (Da)	[M+H^+^] Measured (Da)	RRT Measured	*A. flavus* Biofilm	*A. oryzae* Biofilm	*A. flavus* Submerged	*A. oryzae* Submerged
Pyrazines	Aspergillic acid	C_12_H_20_N_2_O_2_	22,415,248	22,515,975	129,351				
Indole-tetramates	Beta-cyclopiazonic acid	C_20_H_22_N_2_O_3_	33,816,306	35,517,030	109,803				
Indole-tetramates	Cyclopiazonic acid	C_20_H_20_N_2_O_3_	33,614,740	33,715,467	145,486				
Pyrazines	Ferrineaspergillin	C_36_H_57_FeN_6_O_6_	72,536,890	72,637,617	210,616				
Pyrazines	Flavacol	C_12_H_20_N_2_O	20,815,756	20,916,516	125,951				
Indole-tetramates	Speradine A	C_21_H_22_N_2_O_4_	36,615,796	36,716,523	142,600				

(B: biofilm and S: submerged) Metabolite amount quantification according to a heatmap illustration. 
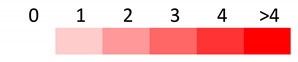

## References

[1] Scholte E.-J., Knols B.G.J., Samson R.A., Takken W. (2004). Entomopathogenic fungi for mosquito control: A review. J. Insect Sci..

[2] Bawin T., Seye F., Boukraa S., Zimmer J.-Y., Delvigne F., Francis F. (2014). La lutte contre les moustiques (Diptera: Culicidae): Diversité des approches et application du contrôle biologique. Can. Entomol..

[3] Bawin T., Seye F., Boukraa S., Zimmer J.-Y., Raharimalala F.N., Zune Q., Ndiaye M., Delvigne F., Francis F. (2016). Production of two entomopathogenic *Aspergillus* species and insecticidal activity against the mosquito *Culex quinquefasciatus* compared to *Metarhizium anisopliae*. Biocontrol Sci. Technol..

[4] Seye F., Bawin T., Boukraa S., Zimmer J.-Y., Ndiaye M., Delvigne F., Francis F. (2014). Pathogenicity of Aspergillus clavatus produced in a fungal biofilm bioreactor toward *Culex quinquefasciatus* (Diptera: Culicidae). J. Pestic. Sci..

[5] WHO-IARC (2002). IARC Monographs on the Evaluation of Carcinogenic Risks to Humans.

[6] Woloshuk C.P., Shim W.B. (2013). Aflatoxins, fumonisins, and trichothecenes: A convergence of knowledge. FEMS Microbiol. Rev..

[7] Abbas H.K., Zablotowicz R.M., Horn B.W., Phillips N.A., Johnson B.J., Jin X. (2011). Comparison of major biocontrol strains of non-aflatoxigenic *Aspergillus flavus* for the reduction of aflatoxins and cyclopiazonic acid in maize. Food Addit. Contam. Part A Chem. Anal. Control Expo. Risk Assess..

[8] Bandyopadhyay R., Ortega-Beltran A., Akande A., Mutegi C., Atehnkeng J., Kaptoge L., Senghor A., Adhikari B., Cotty P. (2016). Biological control of aflatoxins in Africa: Current status and potential challenges in the face of climate change. World Mycotoxin J..

[9] Kagot V., Okoth S., De Boevre M., De Saeger S. (2019). Biocontrol of Aspergillus and Fusarium Mycotoxins in Africa: Benefits and Limitations. Toxins.

[10] Medina M.L., Haynes P.A., Breci L., Francisco W.A. (2005). Analysis of secreted proteins from *Aspergillus flavus*. Proteomics.

[11] Kang D., Son G.H., Park H.M., Kim J., Choi J.N., Kim H.Y., Lee S., Hong S.-B., Lee C.H. (2013). Culture condition-dependent metabolite profiling of Aspergillus fumigatus with antifungal activity. Fungal Boil..

[12] Calvo A.M., Cary J.W. (2015). Association of fungal secondary metabolism and sclerotial biology. Front. Microbiol..

[13] Barrios-González J. (2012). Solid-state fermentation: Physiology of solid medium, its molecular basis and applications. Process Biochem..

[14] Zacchetti B., Wösten H.A., Claessen D. (2018). Multiscale heterogeneity in filamentous microbes. Biotechnol. Adv..

[15] Khalesi M., Zune Q., Telek S., Riveros-Galan D., Verachtert H., Toye D., Gebruers K., Derdelinckx G., Delvigne F. (2014). Fungal biofilm reactor improves the productivity of hydrophobin HFBII. Biochem. Eng. J..

[16] Zune Q., Delepierre A., Gofflot S., Bauwens J., Twizere J.-C., Punt P.J., Francis F., Toye D., Bawin T., Delvigne F. (2015). A fungal biofilm reactor based on metal structured packing improves the quality of a Gla: GFP fusion protein produced by *Aspergillus oryzae*. Appl. Microbiol. Biotechnol..

[17] Tremblay Y.D.N., Hathroubi S., Jacques M. (2014). Bacterial biofilms: Their importance in animal health and public health]. Can. J. Vet. Res..

[18] Biesebeke R.T., Ruijter G., Rahardjo Y.S., Hoogschagen M., Heerikhuisen M., Levin A., A Van Driel K.G., Schutyser M., Dijksterhuis J., Zhu Y. (2002). *Aspergillus oryzae* in solid-state and submerged fermentations. Progress report on a multi-disciplinary project. FEMS Yeast Res..

[19] Van Hartingsveldt W., Mattern I.E., Van Zeijl C.M.J., Pouwels P.H., Hondel C.A.M.J.J.V.D. (1987). Development of a homologous transformation system for Aspergillus niger based on the pyrG gene. Mol. Genet. Genom..

[20] Kakahi F.B., Ly S., Tarayre C., Deschaume O., Bartic C., Wagner P., Compère P., Derdelinckx G., Blecker C., Delvigne F. (2019). Modulation of fungal biofilm physiology and secondary product formation based on physico-chemical surface properties. Bioprocess Biosyst. Eng..

[21] Ly S., Kakahi F.B., Mith H., Phat C., Fifani B., Kenne T., Fauconnier M.-L., Delvigne F. (2019). Engineering Synthetic Microbial Communities through a Selective Biofilm Cultivation Device for the Production of Fermented Beverages. Microorganisms.

[22] OMS (2015). Guidelines for Laboratory and Field Testing of Mosquito Larvicides.

[23] Dagnelie P. (1994). Théorie et Méthodes Statistiques: Applications Agronomiques (Vol. 2).

[24] De Moraes A.M.L., Da Costa G.L., Barcellos M.Z.D.C., De Oliveira R.L., De Oliveira P.C. (2001). The entomopathogenic potential ofAspergillus spp. in mosquitoes vectors of tropical diseases. J. Basic Microbiol..

[25] Zhang P., You Y., Song Y., Wang Y., Zhang L. (2015). First record of *Aspergillus oryzae* (Eurotiales: Trichocomaceae) as an entomopathogenic fungus of the locust, Locusta migratoria (Orthoptera: Acrididae). Biocontrol Sci. Technol..

[26] Vijayan V., Balaraman K. (1991). Metabolites of fungi & actinomycetes active against mosquito larvae. Indian J. Med. Res..

[27] Oda K., Kakizono D., Yamada O., Iefuji H., Akita O., Iwashita K. (2006). Proteomic Analysis of Extracellular Proteins from *Aspergillus oryzae* Grown under Submerged and Solid-State Culture Conditions Proteomic Analysis of Extracellular Proteins from *Aspergillus oryzae* Grown under Submerged and Solid-State Culture Conditi. Appl. Environ. Microbiol..

[28] Hata Y., Ishida H., Ichikawa E., Kawato A., Suginami K., Imayasu S. (1998). Nucleotide sequence of an alternative glucoamylase-encoding gene (glaB) expressed in solid-state culture of Aspergillus oryzae. Gene.

[29] Meurer F., Do H.T., Sadowski G., Held C. (2017). Standard Gibbs energy of metabolic reactions: II. Glucose-6-phosphatase reaction and ATP hydrolysis. Biophys. Chem..

[30] Ortiz-Urquiza A., Riveiro-Miranda L., Santiago-Alvarez C., Quesada-Moraga E. (2010). Insect-toxic secreted proteins and virulence of the entomopathogenic fungus Beauveria bassiana. J. Invertebr. Pathol..

[31] Stalinski R., Laporte F., Després L., Tetreau G. (2016). Alkaline phosphatases are involved in the response ofAedes aegyptilarvae to intoxication withBacillus thuringiensissubsp.israelensis Cry toxins. Environ. Microbiol..

[32] Berthold C.L., Toyota C.G., Richards N.G.J., Lindqvist Y. (2007). Reinvestigation of the Catalytic Mechanism of Formyl-CoA Transferase, a Class III CoA-transferase. J. Boil. Chem..

[33] Mullins E., Starks C.M., Francois J.A., Sael L., Kihara D., Kappock T.J. (2012). Formyl-coenzyme A (CoA): Oxalate CoA-transferase from the acidophile Acetobacter aceti has a distinctive electrostatic surface and inherent acid stability. Protein Sci..

[34] Andrade P., Caldas E. (2015). Aflatoxins in cereals: Worldwide occurrence and dietary risk assessment. World Mycotoxin J..

[35] Blaney B.J., Green P. (1989). Insecticidal Fungal Metabolites: Cyclopiazonic Acid and Kojic Acid Contribute to the Toxicity of “*Aspergillus flavus*” to Sheep Blowfly “Lucilia Cuprina.”.

[36] Geiser D.M., Dorner J.W., Horn B.W., Taylor J.W. (2000). The Phylogenetics of Mycotoxin and Sclerotium Production in *Aspergillus flavus* and *Aspergillus oryzae*. Fungal Genet. Boil..

[37] Frisvad J.C., Skouboe P., Samson R.A. (2005). Taxonomic comparison of three different groups of aflatoxin producers and a new efficient producer of aflatoxin B1, sterigmatocystin and 3-O-methylsterigmatocystin, Aspergillus rambellii sp. nov. Syst. Appl. Microbiol..

[38] Lebar M., Cary J.W., Majumdar R., Carter-Wientjes C.H., Mack B.M., Wei Q., Uka V., De Saeger S., Di Mavungu J.D. (2018). Identification and functional analysis of the aspergillic acid gene cluster in *Aspergillus flavus*. Fungal Genet. Boil..

[39] Nishie K., Cole R., Dorner J. (1985). Toxicity and neuropharmacology of cyclopiazonic acid. Food Chem. Toxicol..

[40] Batt C.A., Tortorello M.L. Encyclopedia of Food Microbiology.

[41] Fremy J.-M. (2009). Évaluation des risques liés à la présence de mycotoxines dans les chaînes alimentaires humaine et animale Rapport final. Afssa.

[42] Tokuoka M., Kikuchi T., Shinohara Y., Koyama A., Iio S.-I., Kubota T., Kobayashi J., Koyama Y., Totsuka A., Shindo H. (2015). Cyclopiazonic acid biosynthesis gene cluster gene cpaM is required for speradine A biosynthesis. Biosci. Biotechnol. Biochem..

[43] Nishimura A., Yoshizako F., Chubachi M. (1997). Purification and Characterization of an Enzyme That Catalyzes Ring Cleavage of Aspergillic Acid, from Tvichoderma koningii ATCC 76666. Biosci. Biotechnol. Biochem..

[44] Macdonald J.C. (1962). Biosynthesis of hydroxyaspergillic acid. J. Boil. Chem..

[45] Micetich R.G., Macdonald J.C. (1965). Biosynthesis of Neoaspergillic and Neohydroxyaspergillic acids. J. Boil. Chem..

[46] Zhu F., Wu J., Chen G., Lu W., Pan J. (2011). Biosynthesis, characterization and biological evalutation of Fe(III) and Cu(II) complexes of neoaspergillic acid, a hydroxamate siderophore produced by co-cultures of two marine-derived mangrove epiphytic fungi. Nat. Prod. Commun..

[47] Arroyo-Manzanares N., Di Mavungu J.D., Uka V., Malysheva S.V., Cary J.W., Ehrlich K.C., Vanhaecke L., Bhatnagar D., De Saeger S. (2015). Use of UHPLC high-resolution Orbitrap mass spectrometry to investigate the genes involved in the production of secondary metabolites in *Aspergillus flavus*. Food Addit. Contam. Part A.

